# The Diagnosis of and Preoperative Planning for Rapidly Progressive Osteoarthritis of the Hip: The Role of Sagittal Spinopelvic Geometry and Anterior Acetabular Wall Deficiency—A Prospective Observational Study

**DOI:** 10.3390/diagnostics15131647

**Published:** 2025-06-27

**Authors:** Andrei Oprișan, Andrei Marian Feier, Sandor Gyorgy Zuh, Octav Marius Russu, Tudor Sorin Pop

**Affiliations:** 1 Doctoral School, George Emil Palade University of Medicine, Pharmacy, Science, and Technology of Targu Mures, 540142 Targu Mures, Romania; andrei.oprisan@umfst.ro; 2 Department of Orthopaedics and Traumatology, Clinical County Hospital of Mureș, 540139 Targu Mures, Romania; sandor.zuh@umfst.ro (S.G.Z.); octav.russu@umfst.ro (O.M.R.); tudor.pop@umfst.ro (T.S.P.); 3 Department M4 Clinical Sciences, Orthopedics and Traumatology I, George Emil Palade University of Medicine, Pharmacy, Science, and Technology of Targu Mures, 540139 Targu Mures, Romania

**Keywords:** rapidly progressive osteoarthritis, spinopelvic alignment, anterior acetabular wall deficiency, advanced imaging techniques, total hip replacement planning

## Abstract

**Background/Objectives**: Rapidly progressive osteoarthritis of the hip (RPOH) has unique diagnostic and surgical challenges due to rapid joint degeneration and acetabular structural alterations. This study aimed to investigate correlations between preoperative spinopelvic geometry and anterior acetabular wall bone stock deficiency in RPOH patients and introduce an advanced imaging measurement techniques for cases with amputated femoral heads. **Methods**: A prospective observational study was conducted that enrolled 85 patients, comprising 40 with unilateral RPOH (Zazgyva Grade II or III) and 45 controls with primary osteoarthritis (OA). Preoperative spino-pelvic parameters (pelvic tilt—PT, sacral slope—SS, lumbar lordosis—LL, and T1 pelvic angle) and acetabular anterior wall characteristics (anterior center edge angle—ACEA, anterior wall index—AWI, and anterior acetabular surface area—AASA) were measured using standardized radiographic and CT imaging protocols, including a new methodology for acetabular center estimation in femoral head-amputated cases. **Results**: Significant differences were identified between RPOH and primary OA patients in the PT (22.5° vs. 18.9°, *p* = 0.032), SS (37.8° vs. 41.1°, *p* = 0.041), T1 pelvic angle (14.3° vs. 11.8°, *p* = 0.018), and anterior center edge angle (25.3° vs. 29.7°, *p* = 0.035). RPOH patients exhibited pronounced spinopelvic misalignment and anterior acetabular deficiencies. **Conclusions**: RPOH is associated with spinopelvic misalignment and anterior acetabular wall deficiency. Accurate preoperative diagnosis imaging and personalized surgical approaches specifically addressing acetabular bone stock deficiencies are mandatory in these cases.

## 1. Introduction

Rapidly progressive osteoarthritis of the hip (RPOH) involves rapid joint degeneration and leads to complex diagnostic and surgical challenges [1]. The incidence of RPOH was previously unclear but has been reported to affect approximately 3% of patients diagnosed with any form of osteoarthritis (OA) [2]. RPOH disproportionately affects elderly patients, particularly women, leading to significant morbidity and reduced quality of life [3]. Standardized assessments of spinopelvic geometry and acetabular bone stock deficiencies could facilitate better surgical planning, reduce variability in outcomes, and guide a more personalized treatment modality. Limited research on RPOH links anterior acetabular wall deficiency to malalignment in spinopelvic alignment and its impact on THA outcomes [2,4]. A common feature of lumbar kyphosis and posterior pelvic tilt is that it decreases anterior acetabular coverage, which can increase the hip weight load and accelerate the morpho-pathological progress of OA [5]. Proper cup anteversion placement is critical for ensuring implant stability and reducing the risk of postoperative dislocation, especially in RPOH cases where anatomical challenges are more pronounced. Anterior acetabular wall deficiency and support for cup placement during THA present unique issues during surgery [6,7]. Positioning the acetabular cup to correct for spinopelvic misalignment can be complex. Misalignment may lead to suboptimal cup anteversion, increasing the risk of implant instability and complications postoperatively [8]. Compared to primary or idiopathic OA, where acetabular structure is typically less compromised, RPOH cases often encounter greater difficulties in achieving stable acetabular cup placement [9]. Currently, no standardized guidelines exist for addressing the challenges of RPOH diagnosis and surgical treatment planning, particularly in patients with significant spinopelvic geometry disturbances and/or a deficient anterior acetabular wall. In patients with advanced RPOH, severe femoral head resorption often makes standard radiographic measurements of anterior coverage infeasible. Some studies have explored spinopelvic alignment in hip OA but none have addressed its interaction with acetabular wall morphology in RPOH, and none have proposed specific CT-based methods for quantifying anterior coverage. This lack of standardized measurement techniques limits diagnostic precision and surgical planning in complex cases. The aim of this observational analysis was to confirm correlations between preoperative spino-pelvic geometry and acetabular bone stock deficiencies. A second aim was to offer understandings into optimizing RPOH diagnosis and THA planning through advanced imaging measurements for cases where the femoral head is already amputated.

## 2. Materials and Methods

### 2.1. Design and Enrollment

**Figure 1:**
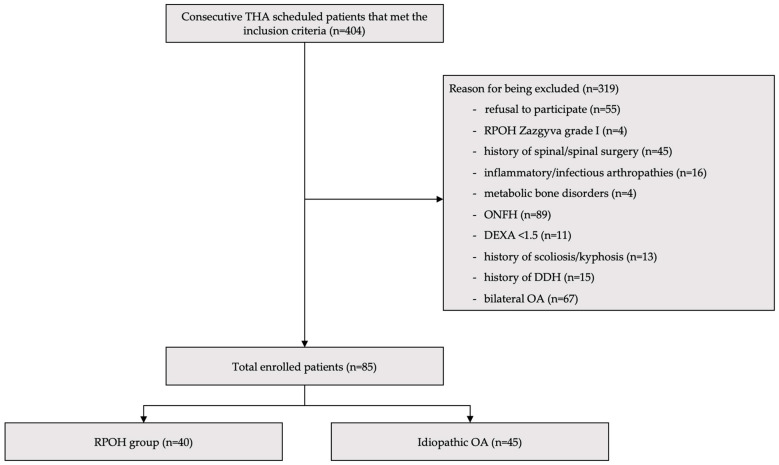
Patient inclusion flowchart. THA—total hip arthroplasty; RPOH—rapidly progressive osteoarthritis of the hip; ONFH—osteonecrosis of the femoral head; DEXA—dual energy x-ray absorptiometry; DDH—developmental dysplasia of the hip; OA—osteoarthritis.

This is a prospective observational study aimed at evaluating the relationship between preoperative spino-pelvic geometry and anterior acetabular deficiency in patients undergoing THA for RPOH. The study was approved by the Institutional Review Board (SCJM21211), and informed consent was collected from all patients by a study nurse. Patient confidentiality was ensured through the anonymization of all data prior to analysis. Unique study identification numbers were assigned to each participant, and all personal identifiers (names, addresses, and contact details) were removed from the dataset, according to recommendations from European Economic Area General Data Protection Regulation 2016/679. Imaging and demographic data were stored on a secure, password protected computer, accessible only to authorized members of the research team. A total of 404 patients who were scheduled for primary THA between October 2022 and October 2024 were included in the study. The final cohort consisted of 40 patients with unilateral RPOH and 45 matched controls with idiopathic/primary OA (Figure 1). The inclusion criteria consisted of patients diagnosed with either RPOH classified as Zazgyva Grade II or III (Table 1) [10] or idiopathic OA.

All patients were at an advanced stage of the disease and were scheduled for primary THA, with complete preoperative imaging available. The exclusion criteria comprised a history of hip or spinal surgery, inflammatory arthropathies, infectious arthritis, metabolic bone disorders, osteonecrosis of the femoral head, hip OA secondary to developmental dysplasia of the hip, and any other conditions potentially affecting acetabular bone stock (DEXA T score of under 1.5) or spinopelvic alignment.

### 2.2. Imaging Analysis Measurements

**Figure 2:**
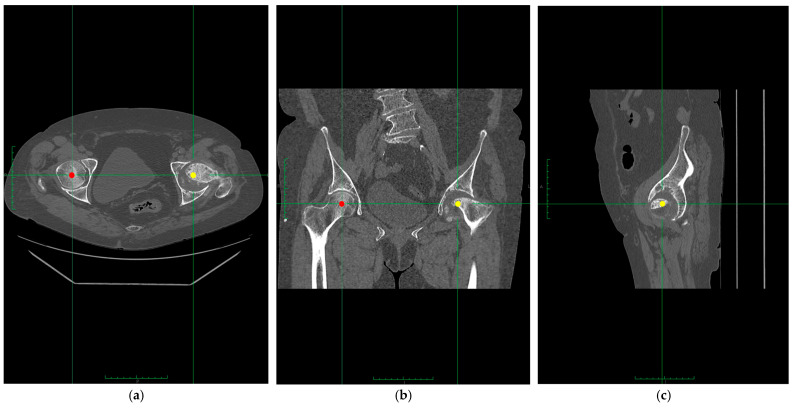
Anterior center edge angle was measured using multiplanar CT reconstruction to accurately determine the center of the acetabulum. Axial (**a**), coronal (**b**), and sagittal (**c**) planes were correlated to establish the acetabular center. In both axial and coronal planes, the healthy contralateral femoral head ((**a**,**b**)—red dot) was used as a reference point, positioning the axes (green line) through the center of the healthy femoral head. By correlating these two planes ((**a**,**b**)—yellow dot), the acetabular center was determined in the sagittal plane, thus allowing ACEA measurement in the absence/resorbed femoral head.

**Figure 3:**
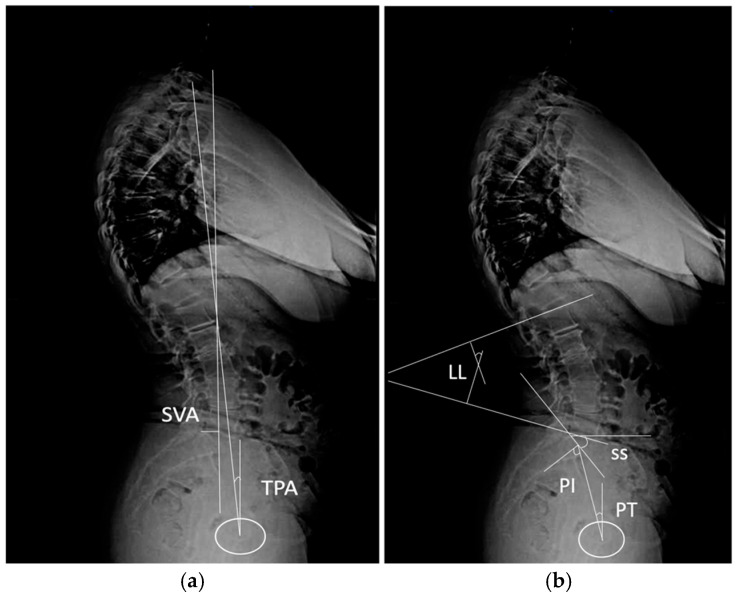
Spino-pelvic and global alignment parameters. (**a**) Spino-pelvic parameters include lumbar lordosis (LL), measured as the angle between the superior endplates of L1 and S1, representing the curvature of the lumbar spine; pelvic incidence (PI), the angle between a perpendicular to the sacral plate at its midpoint and a line connecting this point to the femoral head axis, indicating pelvic morphology; sacral slope (SS), the angle between the sacral plate and a horizontal line, reflecting sacral orientation; and pelvic tilt (PT), the angle between a vertical line and the line connecting the midpoint of the sacral plate to the femoral head axis, representing posterior pelvic rotation. (**b**) Global alignment parameters include the sagittal vertical axis (SVA), defined as the horizontal distance between a vertical line dropped from the center of the C7 vertebral body and the posterior superior corner of the sacral plate, which indicates forward sagittal balance, and the T1 pelvic angle (TPA), the angle between the line connecting the center of the T1 vertebral body to the femoral head axis and the line connecting the femoral head axis to the midpoint of the sacral plate, representing global sagittal alignment independent of pelvic tilt; white circle—femoral head projection.

Preoperative radiographs were independently analyzed by two senior orthopedic surgeons, each with more than 20 years of experience in hip and pelvis surgery. The surgeons evaluated spino-pelvic sagittal misalignment parameters (Figure 2), including pelvic tilt (PT), sacral slope (SS), and lumbar lordosis (LL), as well as acetabular bone stock. All measurements were performed using standardized radiographic protocols [11]. To evaluate the anterior wall of the acetabulum, high-resolution CT scans were used preoperatively with a slice thickness of ≤1 mm. Using 3D reconstruction software (Mimics imaging software v.18, Materialise, Leuven, Belgium), DICOM images were processed to create detailed pelvic models isolating the acetabular region. Anatomical landmarks, including the anterior and posterior acetabular walls, were identified, with the transverse acetabular plane serving as the reference for measurements. All CT scans were acquired in a standardized supine position with the lower limbs in neutral rotation and with a consistent pelvic positioning protocol to minimize variability resulting from tilt or obliquity. During measurement, the pelvic coordinate system was used to realign to anatomical reference planes, and multiplanar CT reformatting was performed to ensure measurements were obtained relative to true anatomical landmarks. Quantitative analysis included the anterior center edge angle (ACEA) measured in the absence of the femoral head (Figure 3) [12], the anterior wall index (AWI) [13], and the anterior and posterior acetabular surface area (AASA, PASA) [14], calculated using integration tools. For cases where the femoral head was absent, we are proposing a new method of measurement.

**Figure 4:**
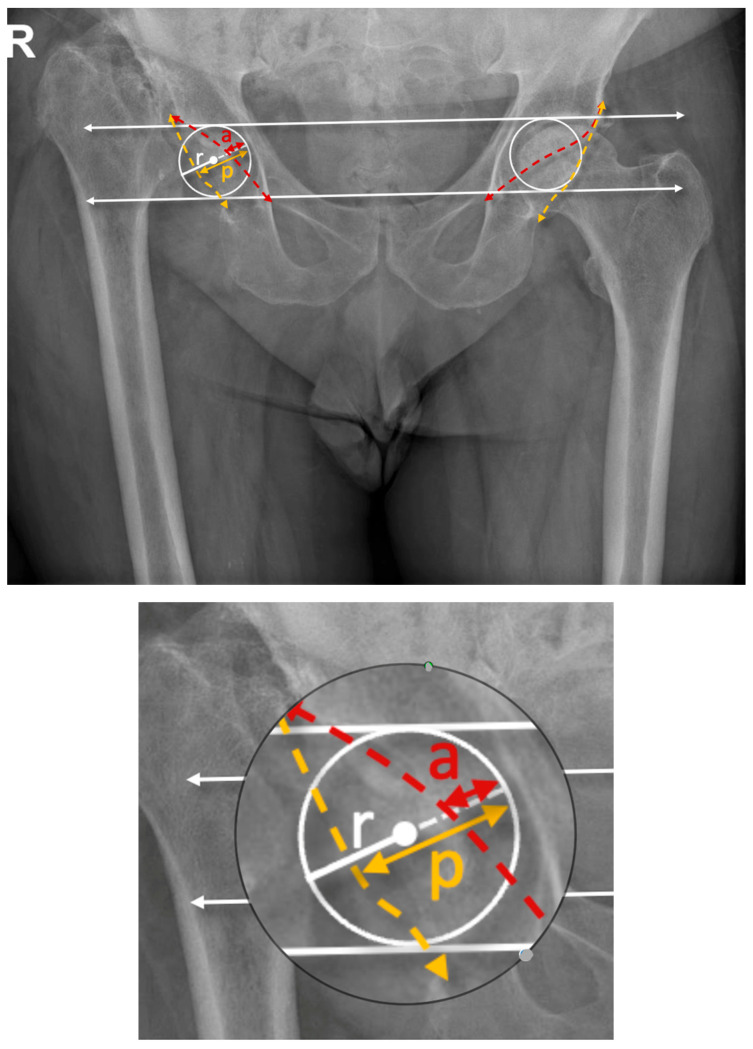
A proposed methodology for anterior wall index measurements in cases with femoral head destruction/amputation (modified after Siebenrock et al. [11]) on a pelvis AP radiograph. The proposed measurement method by our team involves placing a circle at the level of the acetabulum, connecting three anatomical landmarks: the acetabular roof (rim and dome), the medial wall, and a tangent line passing through the acetabular tear drop. The radius (r) and diameter of the circle are afterwards determined. The anterior wall projection (a, red continuous line) is measured along the circle diameter from the medial wall to the intersection of the anterior wall with the circle diameter, while the posterior wall projection (p, yellow continuous line) is measured from the medial wall to the intersection of the posterior wall with the diameter. The anterior wall index (AWI) is calculated as a/r, and the posterior wall index (PWI) as p/r. (red, interrupted line—anterior acetabular wall projection; yellow interrupted line—posterior acetabular wall projection); upper white arrow—acetabular roof, lower white arrow—line connecting radiological teardrops.

The measurement is based on the Siebenrock method [13] and involved placing a circle at the acetabulum level, connecting the acetabular rim, medial wall, and a tangent passing through the acetabular tear drop bilaterally. The anterior wall projection (a) and posterior wall projection (p) were determined along the diameter of the circle, and the indices were calculated as AWI = a/r and PWI = p/r (Figure 4). We proposed a classification for anterior wall deficiencies, when present, with categorization into mild, moderate, or severe based on reductions compared to the normative dataset from the primary OA group (Table 2).

Measurements were performed using Mimics imaging software v.18 (Materialise, Leuven, Belgium). Calibration protocols included the use of a standardized printed phantom to verify dimensional accuracy. All measurements were aligned with the pelvic coordinate system to reduce variability and allow reproducibility of the results. Interobserver and intraobserver reliability were evaluated using intraclass correlation coefficients to ensure consistency and accuracy across all measurements.

### 2.3. Statistical Analysis

Tests were conducted to evaluate the relationship between preoperative spinopelvic geometry and anterior acetabular deficiency. A sample size calculation based on an assumed effect size of 0.5 and a significance level of 0.05 with a power of 80% determined that a minimum of 63 patients were required to detect significant differences or correlations. Descriptive statistics were used to summarize baseline characteristics, demographic data, and imaging parameters. Normality tests and comparisons between groups were performed using *t*-tests or Mann–Whitney U tests for continuous variables and chi-square or Fisher exact tests for categorical data. Correlation analyses, including Pearsons (London, UK), were used to examine the relationships between spino-pelvic geometry and anterior acetabular parameters. A significance threshold of *p* < 0.05 was applied to all tests. Data were analyzed using SPSS (v.27, IBM Corp., Armonk, NY, USA) and R statistical software v4.4.3 (R Foundation for Statistical Computing, Vienna, Austria).

## 3. Results

The study included 85 patients (40 with unilateral RPOH and 45 with idiopathic/primary OA). The mean age was 65.3 ± 8.7 years in the RPOH group and 64.1 ± 9.2 years in the control group (*p* = 0.45). The proportion of female patients was higher in both groups (RPOH: 62.5%, control: 66.7%). BMI was comparable between groups, with a mean of 28.4 ± 3.5 kg/m^2^ in the RPOH group and 27.9 ± 3.2 kg/m^2^ in the control group (*p* = 0.61).

Patients with RPOH exhibited minor demographic differences compared to those with primary OA (Table 3).

Smoking prevalence was higher in the RPOH group (37.5% vs. 26.7%, *p* = 0.353), as was NSAID use (87.5% vs. 73.3%, *p* = 0.173), though these differences were not statistically significant. Intra-articular hip corticosteroid injections were significantly more frequent in the RPOH group (45% vs. 22.2%, *p* = 0.037).

The spino-pelvic geometry of patients with RPOH differed significantly from those with primary OA in some parameters (Table 4).

RPOH patients exhibited a significantly higher pelvic tilt, indicating increased posterior pelvic rotation and a significantly lower sacral slope, reflecting a flatter sacral orientation that could contribute to decreased lumbar lordosis, impaired spinal alignment, and improper femoral head coverage. The T1 pelvic angle (TPA) was significantly higher in the RPOH group and indicated poorer global sagittal alignment, resembling the increased compensatory mechanisms required in RPOH to maintain posture. Although LL, PI, and the SVA did not show statistically significant differences, clinical trends suggest that RPOH patients experienced a slightly reduced lumbar curvature and a more pronounced forward sagittal displacement (SVA: 38.6 ± 9.2 mm vs. 34.5 ± 8.8 mm, *p* = 0.089). RPOH is associated with pronounced alterations in spinopelvic geometry, particularly in compensatory mechanisms like PT and the TPA.

Table 5 is showing significant differences in anterior wall bone stock measurements between RPOH and primary OA patients.

RPOH patients exhibited a significantly smaller anterior coverage angle (25.3 ± 4.1° vs. 29.7 ± 3.8°, *p* = 0.035), indicating reduced anterior acetabular coverage. Although the anterior acetabular surface area was smaller in the RPOH group (18.6 ± 2.5 cm^2^ vs. 21.2 ± 2.9 cm^2^, *p* = 0.062) and the anterior wall index was lower (0.67 ± 0.12 vs. 0.73 ± 0.10, *p* = 0.054), these differences did not reach statistical significance but do suggest a trend toward anterior wall deficiencies or hypoplasia in RPOH. These findings underscored the importance of addressing anterior wall insufficiencies during preoperative planning and surgical interventions in RPOH patients. Table 6 illustrates the interobserver and intraobserver reliability values for spino-pelvic alignment and anterior wall measurements.

## 4. Discussion

This study confirms the significant association between modifications in sagittal spinopelvic alignment and deficient acetabular coverage, particularly in the anterior acetabular wall. The most significant finding of our study was the insufficiency of bone stock in the anterior acetabular wall observed in patients diagnosed with RPOH who also exhibited pronounced spino-pelvic misalignment. Also, a modified method for estimating the femoral head position was described based on cases with amputated femoral head [13]. Patients with hip pathology including developmental dysplasia, OA, and RPOH often also exhibit spinopelvic alterations that impact stability and function [15,16,17]. Increased pelvic obliquity in adolescents with idiopathic scoliosis and hip dysplasia is associated with sacroiliac asymmetry and iliac obliquity [16]. In dysplastic hip OA, pelvic obliquity influenced by subluxation and contractures correlates with poorer functional outcomes and femoral head displacement [18]. Spinopelvic stiffness and abnormal pelvic tilt have also been implicated in late dislocations following THA, underscoring the importance of proper diagnosis, correctly performed preoperative spinopelvic evaluations, and cautious planning [19]. Our analysis did not explicitly adjust for all potential confounders (spinal pathology, pelvic obliquity, prior spinal fusion, or detailed preoperative functional status), but several steps were implemented to minimize their potential influence. Patients with a known history of spinal or hip surgery, inflammatory arthropathies, metabolic bone disorders, or other conditions affecting spinopelvic alignment were excluded. The imaging review included visual screening for pelvic asymmetries and structural deformities, and only patients with complete and standardized imaging were included in the final analysis. We acknowledge that subclinical spinal conditions or variations in compensatory mechanisms were not formally assessed or incorporated into regression models. This remains a limitation of the present study. The absence of multivariate regression modeling, which limits the ability to control for overlapping risk factors and fully adjust for confounders, is a limitation of our observational analysis. This decision was intentional given the exploratory nature of the present study, which focused on identifying structural and imaging-based differences between RPOH and primary OA rather than establishing predictive models. Future studies aiming to define independent risk factors or predictive markers should incorporate multivariate regression approaches or propensity matched analyses for statistical robustness and stronger support of causal inference.

RPOH is associated with increased posterior pelvic tilt and reduced lumbar lordosis, which accelerates joint instability and functional decline [17]. Okamoto et al. found that patients with RPOA had worse patient-reported outcome metrics compared to those with primary OA even after accounting for spinopelvic parameters, suggesting that other factors such as neuromuscular compensation contribute to the observed differences in outcomes and can bias the results [9]. Individual patient factors such as habitual physical activity, muscular strength, and preoperative functional status significantly influence both sagittal spinopelvic alignment and acetabular morphology in RPOH. Higher levels of core and hip extensor muscle strength often maintained in more active patients are correlated with improved lumbar lordosis and reduced posterior pelvic tilt, thereby increasing anterior acetabular coverage during weight bearing [20]. In contrast, reduced functional capacity, as reflected by lower preoperative Harris Hip Scores or timed-up-and-go performance, is associated with compensatory pelvic retroversion and diminished anterior wall support [15]. An elevated body mass index further exacerbates spinopelvic malalignment by increasing axial loading, which may accelerate anterior wall bone loss in RPOH [21]. Comorbidities such as chronic back pain or hip flexor contractures also alter dynamic pelvic rotation and can predispose patients to asymmetric acetabular wear.

Reductions in ACA and AASA are aggravating factors in joint stability and load distribution in patients with RPOH [22]. A decrease in the anterior center edge angle increases stress concentrations on the articular cartilage, accelerating its wear and contributing further to joint instability [23]. In addition to the anatomical and biomechanical factors, we also observed a significantly higher prevalence of intra-articular corticosteroid injections in the RPOH group compared to controls. While our study was not designed to establish a causal relationship, corticosteroids have been implicated in the acceleration of joint degeneration when administered in the context of unrecognized subchondral insufficiency. This association has been highlighted in previous studies and was recently reviewed in detail by our team [17]. It is also possible that patients receiving corticosteroid injections were already experiencing more aggressive disease progression, introducing a confounding effect. Although pharmacologic exposure was not a primary focus of this study, we believe this finding deserves attention and may prompt future research into the potential iatrogenic or contributory role of corticosteroid use in the pathophysiology of RPOH.

Reduced ASA lowers the contact between the femoral head and acetabulum and adds pressure on the remaining cartilage and increases the speed of degeneration [24]. Altered load distribution patterns observed in RPOH, marked by increased peak pressures and stress concentrations, further amplify instability and immediate pain [25]. The acetabular labrum also contributes to stability and labral damage; inversion can also lead to femoral head displacement, rapid joint space narrowing, and subchondral insufficiency fractures [22]. Reductions in the lateral center edge angle correlate with altered cartilage thickness in the weight-bearing zones, reflecting a compensatory response to deficient bony coverage [26]. Comparatively, CT scan analyses in our study included measurements of the AWI, ACA, and AASA and revealed a strong correlation between RPOH and significant deficiencies in anterior wall acetabular bone stock. A necessity of adding advanced preoperative planning techniques for THA should be implemented in this population. More specifically, medialization of the acetabular component should be considered to address the compromised bone stock. If performed correctly, it ensures adequate protrusion and proper sizing for effective primary impaction fixation [27]. Acetabular medialization has important biomechanical implications in RPOH. By shifting the hip center medially, the abductor muscle moment arm is increased, resulting in a reduction in hip joint reaction forces during gait slowing joint degeneration [28,29]. Excessive medialization also shifts the load toward the medial acetabular wall, which can overload the lunate surface and predispose patients to medial wall failure or accelerated cartilage wear, as observed in protrusio acetabuli cases [30,31]. Medialization generally enhances bone support and improves cup coverage, but it does not necessarily increase the primary stability of press-fit components; in fact, over-medialized cups may exhibit greater inducible rotation and compromised initial fixation [32]. Although medialization can mitigate lateral impingement, it may heighten the risk of ischiofemoral impingement, especially when the hip center is medialized beyond 2 mm and the flexion and internal rotation range is reduced, altering wear patterns [33,34]. The degree of acetabular medialization in RPOH should be individualized, balancing reduced joint loading and improved coverage against the risks of medial wall overload and implant instability. This approach improves implant stability and reduces the risk of complications associated with deficient anterior acetabular coverage.

Subgroup comparisons related to the anterior acetabular surface area (AASA, *p* = 0.062) and anterior wall index (AWI, *p* = 0.044) demonstrated marginal *p*-values and wider standard deviations. These results likely reflect the morphological heterogeneity inherent to the RPOH population as well as the limited statistical power associated with subgroup sizes. The consistency in direction across all anterior wall parameters (ACEA, AWI, and AASA) suggests a reproducible anatomical pattern indicative of anterior deficiency. We recognize that these borderline findings should be interpreted with caution, but they remain clinically relevant and provide a foundation for further validation in larger, multicenter studies. CT-based measurements of acetabular geometry present several limitations that can influence clinical decision making and surgical planning. Positional variability during CT scanning and changes in pelvic obliquity and tilt can alter measurements, including acetabular anteversion and AASA, with studies showing that each degree of pelvic obliquity can affect the AA by −0.4° and the AASA by 1.93° [21]. We acknowledge this limitation and underscore the necessity for standardized pelvic positioning during imaging to achieve better measurement accuracy. Despite CT being more reliable than radiographic methods, interobserver and intraobserver variability remains a concern in complex cases. However, in our study, interobserver and intraobserver reliability assessments yielded high consistency across all measurements. Inconsistencies between different software programs used for measurement can impact the assessment of femoral head coverage, leading to variations in morphological classification and treatment planning [35]. Dimensional limitations of 2D-CT compared to 3D-CT also require consideration, as 3D-CT provides superior accuracy [35]. Our findings underscore the potential relevance of spinopelvic alignment and anterior acetabular morphology for preoperative planning in RPOH, and we acknowledge that the current analysis is limited to imaging-based correlations. Surgical outcomes (component positioning accuracy, dislocation rates, implant survivorship, and patient reported functional scores) were not assessed in this study and are being collected. As such, the clinical impact of the identified anatomical variations remains to be validated. This study aimed to provide a morphological foundation for understanding the structural challenges in RPOH in the context of THA planning. The inter- and intraobserver reliability assessments were conducted in a single-center setting by experienced orthopedic surgeons within the same institution. This limits the measurement protocol to broader clinical environments. Future studies should aim to validate these measurements across multiple centers and with a more diverse group of evaluators to confirm reproducibility and external applicability.

## 5. Conclusions

There are significant associations between sagittal spinopelvic misalignment and anterior acetabular wall deficiency in patients with RPOH. These patients exhibit significantly increased pelvic tilt, reduced sacral slope, and a higher T1 pelvic angle compared to patients with primary OA. These anatomical alterations may present challenges in total hip arthroplasty planning and component positioning. While our imaging-based analysis highlights structural considerations that could inform surgical decisions, further outcome-based studies are necessary to determine whether specific strategies translate into improved implant stability and better clinical outcomes.

## Figures and Tables

**Table 1:** Diagnostic criteria and radiologic grading system for rapidly progressive osteoarthritis of the hip [10].

Feature/Symptom	Characteristics for RPOH
Hip pain	Started approx. 3 years ago, variable intensity, worsened in the last 6–9 months
Functional joint mobility	Low/moderate limitation
Osteophytes	Absent or reduced
Geodes	Present in the femoral head and/or acetabulum
Grade	Radiographic Features
I	Partial joint space narrowing No femoral head deformation or ascension
II	Complete disappearance of the joint space Deformed femoral head and acetabulum Ascension of the femoral head ≤ 0.5 cm above the radiologic teardrop
III	Complete disappearance of the joint space Partial osteolysis of the femoral head Ascension of the femoral head > 0.5 cm above the radiologic teardrop

**Table 2:** Classification of anterior wall deficiency based on anterior wall index.

Deficiency	Anterior Wall Index	Description
Mild	0.67–0.70	close to normative values but slightly reduced
Moderate	0.64–0.66	notable reduction, likely affecting coverage and load distribution
Severe	<0.64	marked reduction, indicating significant structural compromise

**Table 3:** Demographic and patient characteristics.

Characteristic	RPOH	Primary OA
Female, no (%)	25 (62.5)	30 (66.7)
Weight, kg, mean ± SD	78.5 ± 9.8	76.3 ± 8.7
Age, years, mean ± SD	65.3 ± 8.7	64.1 ± 9.2
Smoker, >1 year, yes, (%)	15 (37.5)	12 (26.7)
Oral NSAIDs used, yes (%)	35 (87.5)	33 (73.3)
Intraarticular corticosteroid injections < 6 mo., yes, no (%)	18 (45)	10 (22.2)

NSAIDs—nonsteroidal anti-inflammatory drugs; mo—months.

**Table 4:** Quantitative data for spino-pelvic geometry.

Imaging Feature	RPOH (Mean ± SD)	Primary OA (Mean ± SD)	*p* Value
Pelvic tilt, °	22.5 ± 4.3	18.9 ± 3.8	0.032
Sacral slope, °	37.8 ± 6.2	41.1 ± 5.7	0.041
Lumbar lordosis, °	47.5 ± 8.6	50.2 ± 7.4	0.148
Pelvic incidence, °	56.3 ± 7.1	58.5 ± 6.9	0.247
Sagittal vertical axis, mm	38.6 ± 9.2	34.5 ± 8.8	0.089
T1 pelvic angle, °	14.3 ± 2.9	11.8 ± 3.2	0.018

°—degrees; mm—millimeters; SD—standard deviation.

**Table 5:** Quantitative data for acetabular anterior wall bone stock measurements.

Anterior Wall Characteristics	RPOH (Mean ± SD)	Primary OA (Mean ± SD)	*p* Value
Anterior center edge angle, °	25.3 ± 4.1	29.7 ± 3.8	0.035
Anterior acetabular surface area, cm^2^	19.6 ± 2.5	21.2 ± 2.9	0.062
Posterior acetabular surface area, cm^2^	25.6 ± 2.2	27.3 ± 2.5	0.069
Anterior wall index	0.69 ± 0.12	0.73 ± 0.10	0.044

°—degrees; cm^2^—centimeter square; SD—standard deviation.

**Table 6:** Interobserver and intraobserver reliability for spino-pelvic alignment and anterior wall measurements.

Parameter	Interobserver ICC	Intraobserver ICC
Pelvic tilt	0.91 (0.88–0.94)	0.94 (0.92–0.96)
Sacral slope	0.89 (0.85–0.92)	0.93 (0.90–0.95)
Lumbar lordosis	0.87 (0.83–0.90)	0.92 (0.89–0.94)
Sagittal vertical axis	0.88 (0.84–0.91)	0.91 (0.88–0.93)
T1 Pelvic Angle	0.90 (0.86–0.93)	0.93 (0.91–0.95)
Anterior Center Edge Angle	0.89 (0.85–0.92)	0.92 (0.89–0.94)
Anterior/Posterior Acetabular Surface Area (amputated head, RPOH)	0.88 (0.84–0.91)	0.91 (0.88–0.93)
Anterior Wall Index (amputated head, RPOH)	0.87 (0.83–0.90)	0.90 (0.87–0.93)
Anterior/Posterior Acetabular Surface Area (primary OA)	0.84 (0.81–0.90)	0.90 (0.87–0.94)
Anterior Wall Index (primary OA)	0.88 (0.84–0.91)	0.88 (0.86–0.91)

## Data Availability

The data presented in this study are available on request from the corresponding author. Data are not publicly available due to privacy or ethical restrictions.
